# Hypothalamic neurohormones and immune responses

**DOI:** 10.3389/fnint.2013.00056

**Published:** 2013-08-13

**Authors:** J. Luis Quintanar, Irene Guzmán-Soto

**Affiliations:** Laboratory of Neurophysiology, Department of Physiology and Pharmacology, Centro de Ciencias Básicas, Universidad Autónoma de AguascalientesAguascalientes, México

**Keywords:** TRH, CRH, CRF, GnRH, LHRH, immune system, extrapituitary, receptors

## Abstract

The aim of this review is to provide a comprehensive examination of the current literature describing the neural-immune interactions, with emphasis on the most recent findings of the effects of neurohormones on immune system. Particularly, the role of hypothalamic hormones such as Thyrotropin-releasing hormone (TRH), Corticotropin-releasing hormone (CRH) and Gonadotropin-releasing hormone (GnRH). In the past few years, interest has been raised in extrapituitary actions of these neurohormones due to their receptors have been found in many non-pituitary tissues. Also, the receptors are present in immune cells, suggesting an autocrine or paracrine role within the immune system. In general, these neurohormones have been reported to exert immunomodulatory effects on cell proliferation, immune mediators release and cell function. The implications of these findings in understanding the network of hypothalamic neuropeptides and immune system are discussed.

## Introduction

The neurosecretion in the hypothalamus can be traced back to the work of Scharrer and Scharrer (1940). Posterior studies by Harris specified that the hypothalamic substances secreted into the portal vessels were pituitary specific and propose the concept of “releasing factors” whose purpose was to initiate a cascade of events resulting in the release of peripherally active hormones (Harris, 1948).

The discovery and chemical characterization of the first identified hypothalamic releasing factor by Burgus et al. (1969) and Boler et al. (1969) provided ultimate confirmation for the founding principles of neuroendocrinology and was followed by the discovery of other peptide-releasing factors (Nillni and Sevarino, 1999).

## Thyrotropin-Releasing Hormone (TRH)

TRH, the smallest known peptide hypophyseotropic hormone, is a tripeptide (pGlu-His-Pro-NH_2_; where pGlu stands for pyroglutamic acid) that is released from the hypothalamus and transported via the portal vascular system to the anterior pituitary where it stimulates the release of thyroid-stimulating hormone (TSH) and prolactin from the anterior pituitary (Matre et al., 2003).

TRH it is also known as thyrotropin-releasing factor (TRF), thyroliberin or protirelin (Khomane et al., 2011) and was the first chemically defined hypophyseotropic hormone (Bílek et al., 2011).

### Synthesis

This small tripeptide (362.42 Da) is indeed processed from a larger precursor protein, prepro-TRH (ppTRH), through now classic cleavage mechanisms, C-terminal amidation, and cyclization of the N-terminal Gln to pGlu (Guillemin, 2005).

The rat ppTRH is a 29 kDa polypeptide composed of 255 amino acids. The rat precursor contains an N-terminal 25-amino-acid leader sequence, five copies of the TRH progenitor sequence Gln-His-Pro-Gly flanked by paired basic amino acids (Lys-Arg or Arg-Arg), four non-TRH peptides lying between the TRH progenitors, an N-terminal flanking peptide, and a C-terminal flanking peptide. Rats and mice have five Gln-His-Pro-Gly TRH progenitor sequences, whereas humans have six TRH sequences (Chiamolera and Wondisford, 2009).

### TRH expression regulation

The maintenance of euthyroidism is dependent on a highly regulated balance of neuropeptides and neurotransmitters, where the dominant positive hypothalamic control for TSH is TRH, and the principal feedback control is through thyroid hormones (THs) (Nillni and Sevarino, 1999).

THs exert powerful feedback inhibition over the TRH response system by inhibiting TRH synthesis and processing in TRH neurons in the paraventricular region of the hypothalamus and decreasing TRH receptors (TRHR) and responses in the pituitary gland (Hinkle et al., 2012).

TRH gene expression is also regulated by temperature, food intake, and stress. Thus the TRH neuron is well positioned to integrate information about the environment as well as circulating TH levels and ultimately affect metabolism in response to these physiological changes (Chiamolera and Wondisford, 2009).

### Half-life

The short half-life (4–5 mins) of TRH is most likely due to rapid degradation of the TRH at both the carboxy (COOH) and amino (NH_2_) termini. Cleavage of the pyroglutamyl moiety of TRH by enzymes like aminopeptidases and deamidases causes formation of the metabolite histidyl proline diketopiperazine (ClycoHistine-Proline, CHP) while its deamidation results in the formation of the free acid, that is, TRH-OH (Khomane et al., 2011).

### TRHR

The TRHR is a member of a large family of transmembrane proteins (G-protein-coupled receptors, GPCRs) for which an interaction with an intracellular G protein is a critical part of the signal transduction pathway mediated by the receptor. It is thought that all GPCRs have a common tertiary structure composed of seven transmembrane helices. The topology of these membrane-bound proteins is defined by an extracellular amino terminus and an intracellular COOH terminus. Consequently, the helices are connected by intracellular-extracellular loops. Colson et al. (1998) suggested that TRH initially interacts with residues within the extracellular loops and then moves into the transmembrane binding pocket, aided by the motion of the loops.

A single TRHR gene has been found in humans and higher mammals and two genes encoding homologous receptors, TRHR1 and TRHR2, in rodents (Sun et al., 2003). TRHR1 predominates in the anterior pituitary gland while both TRHR1 and TRHR2 are found in rodent central nervous system (CNS) (Hinkle et al., 2012).

### Extrahypothalamic and Extrapituitary location of TRH and TRHR respectively

Originally discovered in the hypothalamus, consistent with its classical role as a hypothalamic hypophysiotrophic factor, TRH and TRHR are now known to be distributed throughout the central and peripheral nervous system (PNS) and are found extensively in extrahypothalamic brain structures and in other organs and tissues (Sun et al., 2003; Kamath et al., 2009).

### TRH outside the pituitary zone

TRH has been located in several brain areas other than the paraventricular nucleus as well as in non-CNS tissues:

- Pancreatic β-cells (Engler et al., 1981; Kawano et al., 1983).
- The whole gastrointestinal tract where it may modulate contractions (Morley, 1979; Engler et al., 1981).
- The genitourinary system. TRH is found in rat, canine and human prostate, seminal vesicles, ovary, testis, Leydig cells and epididymis (Pekary et al., 1980, 1983a,b; Feng et al., 1992).
- The presence of TRH in rat, human, porcine and bovine retina has been reported (Schaeffer et al., 1977; Martino et al., 1980a,b).
- Shambaugh et al. (1979) identified TRH activity in placental extracts by specific radioimmunoassay test (RIA).
- TRH has been found as part of immune system tissues and blood elements (Matre et al., 2003). This topic will be discussed in depth in the following sections.

### TRHR outside the hypothalamic zone

Besides the well-known TRHR location in the pituitary zone, TRH binding sites have been described throughout the central and PNS and in non-CNS tissues including:

- Uterus, ovary and testis where TRHR mRNA levels appear to be relatively high (Fukusumi et al., 1995; Montagne et al., 1999).
- Wang et al. (1997) reported that small intestine epithelial cells were found to express receptors for TRH and to be a primary source of intestine-derived TSH.
- TRHR is also found in retina (Satoh et al., 1993) and adrenal gland (Montagne et al., 1999).

Furthermore, TRHR expression in organs and cells of the immune system has been confirmed:

- Expression of TRHR mRNA and TRHR in lymphoid tissues has been found: Thymus, mesenteric lymph nodes and spleen (Raiden et al., 1995; Mellado et al., 1999).
- Thymus expression of the TRHR has been reported by several authors (Fukusumi et al., 1995; Mellado et al., 1999; Montagne et al., 1999; Matre et al., 2003).
- Expression analysis in rat demonstrated the presence of TRHR mRNA in bone marrow (Montagne et al., 1999; Matre et al., 2003) and in the rat natural-killer cell line RNK-16 (Matre et al., 2003). This expression was shown to be functional as evidenced by TRH binding to surface receptors on thymocytes and RNK-16 cells.
- Analysis of human peripheral blood lymphocyte (Raiden et al., 1995; Mellado et al., 1999) and tonsil-derived leukocyte populations showed TRHR expression in non-activated and phytohemagglutinin-activated T and B cells (Mellado et al., 1999).

The widespread distribution of TRH and the TRHR within and outside the CNS supports a diverse range of roles for this molecule, roles likely to involve many functions outside of the traditional hypothalamic-pituitary-thyroid (HPT) axis (Nillni and Sevarino, 1999).

### TRH functions

TRH indirectly stimulates TH biosynthesis and release. Thus, TRH is central in regulating the HPT axis. TRH influences the release of other hormones, including prolactin, growth hormone, vasopressin, insulin, and the classic neurotransmitters noradrenaline and adrenaline (Nillni and Sevarino, 1999).

### Extrahypophysiotropic functions of TRH

More than two-thirds of immunoreactive-TRH in the brain is found outside of the traditional “thyrotrophic zone” of the hypothalamus. This extrahypophysiotropic TRH is believed to function as a neuromodulator of known neurotransmitters. Indeed, it might act as a neurotransmitter itself; it is present in secretory granules whose exocytosis is responsive to membrane depolarization, it acts through specific receptors that are widely distributed throughout the CNS, and it is rapidly cleared through specific catabolic pathways. In several areas of the brain, TRH is colocalized with other neurotransmitters and/or neuromodulators (Nillni and Sevarino, 1999).

These effects of TRH have been demonstrated using TRH and/or TRH analogs. Available TRH analogs have higher affinities for the TRHR, longer half-lives, etc. (Engel et al., 2006; Khomane et al., 2011; Monga et al., 2011).

Beside endocrine functions, TRH also possesses several neuropharmacological effects like cerebral nerve activation (stimulation of motor function), effects on sympathetic nervous system (elevation of blood pressure and stimulation of respiratory), effects on spinal function (stimulation of spinal motor neuron), effects on CNS (antidepressant activity) and peripheral actions (suppression of gastric acid secretion, stimulation of glucagon secretion). These CNS-mediated effects provide the rationale for the use of TRH in the treatment of brain/spinal injury and certain CNS disorders, including schizophrenia, Alzheimer’s disease, epilepsy, amyotrophic lateral sclerosis, Parkinson’s disease and depression (Khomane et al., 2011).

### TRH and immune system

Two-way communication between the immune and neuroendocrine systems is now well recognized. These interactions are mediated by cytokines, neuropeptides, hormones, other soluble factors and their receptors (Grasso et al., 1998).

As mentioned above, cells of the immune system have been found to contain receptors for neuroendocrine hormones and can also be considered a source of pituitary and hypothalamic peptides (Matre et al., 2003).

### Immune response to TRH administration

#### Proliferation

##### Induction

Acute and chronic administration of TRH enhances the proliferation of splenic and thymic lymphocytes in rats (Pawlikowski et al., 1992; Raiden et al., 1995; Winczyk and Pawlikowski, 2000). In fact, it has been reported that TRH promotes thymic reconstitution in mice with anterior hypothalamic area lesions (Lesnikov et al., 1991). According with these data, it was also reported that TRH enhances the *in vitro* plaque-forming cell response (Kruger et al., 1989), antagonizes the dexometasone-induced thymocyte and splenocyte suppression (Winczyk and Pawlikowski, 2000) and administration in humans led to increased secretion of IL-2 into the blood (Komorowski et al., 1993).

##### Suppression

It has also been reported that TRH significantly inhibits monocyte activity (Lersch et al., 1989). The experimental work of Kunert-Radek et al. (1991) addressed the proliferation of murine splenocytes *in vitro* considering the ^3^H-thymidine incorporation into splenocyte DNA as an index of proliferation. They found that TRH suppressed the proliferation of splenocytes.

TRH has the capability to modulate the natural cell-mediated cytotoxicity. To try it out, TRH was added to the feed of White Leghorn male chicks and peripheral blood lymphocytes were cultured *in vitro* with or without different mitogens (Phytohaemagglutinin-A (PHA), ConA, or Lipopolysaccharide (LPS)), and the culture supernatants were tested for the presence of lymphokines. Results showed that the supernatant from *in vivo* 5 ppm TRH-treated lymphocytes significantly suppressed the natural cell-mediated cytotoxicity (Haddad and Mashaly, 1992).

Recently was published that oral administration of TRH in mice with experimental autoimmune encephalomyelitis (EAE), an animal model of multiple sclerosis, reduces the spinal cord inflammatory foci without increased frequency of regulatory T cells (Treg) in spleen (Brod and Bauer, 2013).

#### Induces TSH production by immune cells

At very low concentrations, TRH induces splenocyte production of TSH *in vitro* (Kruger et al., 1989).

Peripheral blood mononuclear cells (PBMC), rat splenocytes and transformed T cell lines produce TSH in response to TRH (Harbour et al., 1989; Raiden et al., 1995).

#### Induces immune mediators production

PBMC, rat splenocytes and transformed T cell lines can enhance or modulate the *in vitro* antibody response in response to TRH (Kruger et al., 1989; Hart et al., 1990).

Koshida and Kotake (1993) investigated the role of TRH on the superoxide anion (O_2_^−^) production of rabbit peritoneal macrophages. Their results showed that TRH acts on macrophages and suggest that TRH possesses the priming action of O_2_^−^ release in response to the chemotactic peptide N-formyl-methionyl-leucyl-phenylalanine, and opsonized zymosan. Authors suggest that if this type of priming exists *in vivo* there should be a role to play in an inflammation process.

*In vitro* studies have shown that a TRH fixation occurs at the level of the human polymorphonuclear neutrophil, which suggests putative membrane receptor (s) for the hypothalamic hormone. The correspondent *in vivo* analyses demonstrated that after TRH administration, enzyme modifications (myeloperoxydase, alkaline phosphatase) and metabolism changes (PAS, Sudan black) happen, showing a functional activation of that blood cell (Blum et al., 1976). These *in vivo* effects could be due to TRH is being directly attached to its receptor on neutrophils or an indirect effect is being mediated by TSH or THs.

Intravenous bolus of TRH in normoprolactinemic women increases plasma gamma interferon (IFN-*γ*) levels 45 mins after administration. In order to investigate a possible direct action of TRH on immune cells, the authors also examined the effects of this hypothalamic peptide on IFN-**γ** production by PBMCs stimulated *in vitro* with suboptimal concentration of bacterial superantigen staphylococcal enterotoxin A or concanavalin A (ConA), collected before the intravenous administration of TRH. The *in vitro* findings showed a directly enhanced IFN-**γ** production by activated lymphocytes (Grasso et al., 1998).

Furthermore, splenic and CNS lymphocytes showed significant decrease in levels of profile 17 helper t cells (T_H_17) and T_H_1 cytokines: Interleukin-17 (IL-17), tumor necrosis factor (TNF-*α*) and IL-2 respectively, after oral administration of TRH in EAE mice. This treatment also increased CNS lymphocyte production of T_H_2 cytokines, in particular IL-13 (Brod and Bauer, 2013). Thus, oral TRH shows a unique pattern of immunomodulation for an ingested neuropeptide although the poor oral bioavailability associated with TRH and some of its analogs (Khomane et al., 2011).

#### TRH expression response against immune system challenges

It is known that high concentrations of TRH seem to decrease the production of antibodies against sheep red blood cells (SRBC) (Kukaĭn et al., 1982).

Pérez Castro et al. (1999) investigated whether TRH mRNA levels in the hypothalamus are altered after SRBC (a T cell-dependent antigen) immunization. An increased level of TRH mRNA detected by Northern blot autoradiography was observed at 4, 6, and 24 h post immunization in SRBC-injected animals.

The SRBC-induced effects on TRH mRNA levels are opposite to those induced by LPS, a T cell-independent response through macrophage and B lymphocyte activation. LPS induces a decrease in hypothalamic TRH mRNA levels that is observed at 24 h and more pronounced at 4 h (Pérez Castro et al., 1999).

As we can see, TRH effects could seem controversial but is important to consider that interactions between the CNS, the endocrine system and the immune system are mediated at multiple levels. These mediators include secreted chemical messengers such as hormones, cytokines, neurotransmitters and neuropeptides acting directly or via the nervous system.

Evidence indicates interactions at the level of receptors (e.g., the presence of neuroendocrine peptide receptors on immune cells), at the level of secretory function (e.g., the synthesis and secretion of neuroendocrine peptides by immune cells), and at the level of signal transduction (Kamath et al., 2009). Integration of these complex interactions in a single route would be inaccurate, for this reason, the most wise conclusion could be consider the TRH-immune system homeostatic hypothesis proposed by Gary et al. (2003) and Kamath et al. (2009), which states that TRH mediated mechanisms respond to many elements of the immune system and affect them in ways that tend to maintain or restore homeostasis.

**Figure 1:**
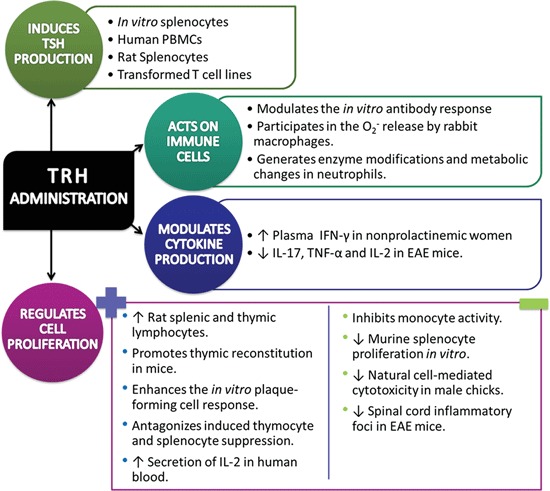
**Immune response against Thyrotropin-Releasing Hormone/Thyrotropin-Releasing Hormone agonist administration in a diversity of normal and pathological conditions (↑= increase, ↓= decrease).**

The effects of TRH administration on immune system are summarized in Figure 1.

### Clinical relevance and future directions

Studies on the effects of TRH *in vitro* hardly can be extrapolated to *in vivo* conditions. The direct effect of TRH on the immune system provides a modulatory physiological role. However, pharmacological administration in humans and in experimental animals involves two pathways impact (1) through stimulation of TSH secretion and therefore of THs and (2) the direct effect on the immune system. TRH administration may affect these two ways and with addition or synergy effects. One possibility to explore the direct mode might be using hypophysectomized or thyroidectomized animals to avoid endocrine influence. According to the studies described above, it is possible to consider the possibility that clinical treatment with TRH may improve the immune response in patients with immune deficiencies or reduced response in autoimmune diseases such as multiple sclerosis or lupus erythematosus. Such expectations are a challenge to be explored in the future.

## Corticotropin-Releasing Hormone (CRH)

CRH or Corticotropin-releasing factor (CRF) was characterized, purified and synthesized in 1981 by Vale. The composition of this 41 amino-acid peptide is different from one species to another. Its principal origin in the brain are the neurons from paraventricular nucleus of the hypothalamus. By the portal hypothalamic-pituitary vessels, it reaches the anterior pituitary lobe where it stimulates the corticotroph cells (Raux-Demay and Girard, 1985; Emeric-Sauval, 1986). The plasma half-life of CRH in humans is 4 mins (Schürmeyer et al., 1984). The action of CRH on adrenocorticotrophic hormone (ACTH) secretion is predominant, but it acts in relation with arginine-vasopressin and potentially with angiotensin and catecholamines. Adrenal steroids (glucocorticoids) act on feedback control of CRH secretion in the hypothalamus and also on pituitary corticotrophs. CRH has shown to be an important regulator of the endocrine stress response, and it is now known to play a role in diverse functions in the homeostatic balance, important in mobilization of resources and behaviors during stress (Bale and Vale, 2004), it is involved in the regulation of food intake and satiety, as well as gastrointestinal tract motility, vascular tone, and development, and also acoustic and cardiac function (Heinrichs et al., 1992; Koob and Heinrichs, 1999; Maillot et al., 2000; Inoue et al., 2003; Janssen and Kozicz, 2013).

### CRH Receptor (CRHR)

High-affinity CRHRs which mediate the actions of the hypothalamic peptide on ACTH release, have been principal identified in the anterior pituitary gland. Interaction of the pituitary receptor by CRH agonists stimulates ACTH release. CRH exerts its effects by binding to specific cell surface receptors, of which two receptor subtypes have been reported, CRH-R1 and CRH-R2. These receptors share high sequence homology and belong to the family of seven transmembrane GPCRs (Vaughan et al., 1995; Lewis et al., 2001).

Actions of this peptide are initiated by binding and activating GPCRs and subsequently the activation of adenylate cyclase and cyclic adenosine monophosphate dependent protein kinase (Grammatopoulos and Chrousos, 2002). In the regulation of ACTH secretion, the effects of CRH on the corticotroph are integrated with the stimulatory actions of cyclic adenosine monophosphate and the inhibitory effects of glucocorticoids. Following adrenalectomy, the progressive elevation of plasma ACTH levels is accompanied by a concomitant decrease in pituitary CRHRs (down-regulation) (Wynn et al., 1985; Aguilera et al., 1986).

#### Extrapituitary expression of CRHR

CRH-R1 is a 415 amino acid peptide and has a wide-spread expression in stress-related areas located in the CNS (Justice et al., 2008; Kuhne et al., 2012). Outside the CNS, the CRH-R1 is expressed in the adrenal gland, gastrointestinal tissue, ovary, testis, skin, thymus and spleen (Dufau et al., 1993; Nappi and Rivest, 1995; Slominski et al., 1995; Baigent and Lowry, 2000; Müller et al., 2001; Chatzaki et al., 2004). CRH-R2 is a 397–437 amino acid protein and it is abundantly expressed in the CNS (Korosi et al., 2007; Justice et al., 2008; Kuhne et al., 2012). In the periphery, the receptor has been identified in the retina, heart, skeletal muscle, vasculature, adrenal gland, and gastrointestinal tissue (Lovenberg et al., 1995; Müller et al., 2001; Chatzaki et al., 2004).

Binding sites for CRH have been reported on adherent splenocytes (Webster et al., 1990), monocyte-macrophages (Audhya et al., 1991) and T lymphocytes (Singh and Fudenberg, 1988). CRH-R1 and CRH-R2 expression was described in granulocytes and lymphocytes in inflamed tissues (Mousa et al., 2003). CRH-R1 mRNA expression had been reported in the human leukemic mast cell line (Theoharides et al., 1998) and mast cells were reported to express immunoreactive CRH-R1 in inflamed human synovial tissue (McEvoy et al., 2001).

### CRH effects on CNS and PNS

Independently of anterior pituitary, CRH has effects within of nervous system. The central administration of CRH produces hypertension, tachycardia and an elevated oxygen consumption. CRF acts within the brain to increase plasma concentrations of noradrenaline and adrenaline resulting in increased plasma concentrations of glucagon and in hyperglycemia. Likewise, also acts increasing the plasma concentrations of vasopressin. CRH induces a reduction in food intake and increases grooming behavior and locomotor activity. The physiological significance of the peripheral actions of CRH on various organ systems is unknown. Intravenous administration of CRH reduces gastric acid secretion, gastric emptying and blood pressure but increases heart rate, venous return to the heart, mesenteric and aortic blood flow and pancreatic bicarbonate and protein secretions (Lenz, 1987; Chen et al., 2007).

### CRH and immune system

The cellular components of the immune-inflammatory response include leukocytes, such as monocytes–macrophages, polymorphonuclear neutrophils, eosinophils and basophils, platelets, dentritic cells, mast cells, epithelial and endothelial cells, and fibroblasts which belong to the innate immune system, as well as lymphocytes T, B and natural killer (NK) which are included in the adaptive immune system. These cells cooperate using molecular signals, including ILs, colony-stimulating factors, TNF, IFNs, transforming growth factor, and chemokines, vasoactive amines (histamine and serotonine), plasma proteases (kinine and complement systems), arachidonic acid metabolites (prostaglandins, leukotrienes, and lipoxins), platelet-activating factor, nitric oxide, and neuropeptides (Mastorakos et al., 2006).

The majority of *in vivo* studies suggest that direct stimulation of hypothalamic CRH by cytokines is the principal route by which immune activation stimulates the hypothalamic–pituitary–adrenal (HPA) axis (Besedovsky et al., 1986; Harbuz et al., 1992) and this process is prostaglandin-dependent. Thus, experimental evidence suggests that CRH may modulate the immune and inflammatory responses via two pathways: an anti-inflammatory one operated by centrally released CRH, most likely through stimulation of glucocorticoid and catecholamine release, and one pro-inflammatory, through direct action of peripherally secreted CRH (Karalis et al., 1997).

#### CRH in immune cells

##### Mast cells

Mast cells are critical for allergic reactions due to their secretion of numerous vasoactive molecules and cytokines (Kobayashi et al., 2000). Evidence indicates that mast cells are also involved in innate and acquired immunity (Marone et al., 2002), as well as in neuroinflammatory conditions such as those affected by stress (Theoharides and Cochrane, 2004). Mast cells secrete numerous pro-inflammatory cytokines, such as IL-6, IL-8, and TNF-*α* (Kobayashi et al., 2000; Marone et al., 2002), and secretion of vascular endothelial growth factor (VEGF) by CRH stimulation (Cao et al., 2005). Interestingly, human mast cells were shown to synthesize and secrete large amounts of CRH, suggesting that this peptide could regulate mast cells through CRH receptor in an autocrine manner. Secretion of CRH from mast cells could also be triggered by pro-inflammatory molecules that are released during the initial phases of inflammation (Kempuraj et al., 2004). CRH causes mast cell degranulation in human skin, releasing great amounts of histamine, which appears to be the principal mediator of the vasodilatory effects of CRH (Wright, 2003).

##### Mononuclear cells

In mononuclear cells, it has been reported that CRH stimulates immune functions, proliferation of lymphocytes both in the absence and presence of T cell mitogens, and the expression of IL-2R antigen on T cells (Singh, 1989; Singh et al., 1990). It has also been reported the presence of binding sites for CRH on monocytes, T and B lymphocytes and thymus (Singh and Fudenberg, 1988). The CRH is also a stimulator of production of two interleukins, IL-1 and IL-2. IL-1 has been shown to stimulate the secretion of CRH by hypothalamic cells (Sapolsky et al., 1987) and CRH stimulates the production of IL-1 and IL-2, suggesting the presence of a complete network between the neuroendocrine system and the immune system (Singh and Leu, 1990).

The release of CRH plays a role in modulating NK activity following stress also implies that CRH in the brain may be involved in the functional population of lymphocytes. Studies have been conducted to analyze the effect of CRF on the reduction of several cellular immune measures such as splenic and peripheral blood NK activity and lymphocyte responses to mitogenic stimulation (Strausbaug and Irwin, 1992; Boyadjieva et al., 2006). Other observations also indicate that central doses of CRH slow the kinetics and reduce the level of IgG response to a specific T-cell-dependent antigen (Irwin, 1993). The administration of central CRH antagonist induces suppression of NK cytotoxicity. In contrast, peripheral application has no effect on CRH modulation of immunity (Irwin et al., 1987).

Other studies have shown CRH to inhibit IFN-**γ** secretion (Angioni et al., 1993). IL-2-induced splenocyte proliferation as well as LPS-mediated IL-1 and IL-6 production by peripheral blood monocytes (Hagan et al., 1992).

The effects of CRH were almost exclusively examined in the mononuclear cells, however in other studies has been reported that polymorphonuclear cells (neutrophils), express higher levels of CRH-R1 than mononuclear immune cells in mouse (Radulovic et al., 1999). It has been shown that CRH inhibits the secretion of IL-1β from mature neutrophils purified from spleens of mice injected with LPS (Radulovic and Spiess, 2001).

It has been described the CRH presence in macrophages (Brouxhon et al., 1998). Results showed that CRH could be detected in normal peritoneal macrophages and the levels increased significantly after stimulation with LPS (Wang et al., 2012). The role of CRH may be immunomodulatory, however the effects remains to be studied.

On other hand, studies shown that cluster of differentiation (CD14+) group of cell surface marker proteins, the CRH can activate CD14+ cells to produce TNF-*α* cells and compromise endothelial barrier function by inducing apoptosis of the endothelial cells (Song et al., 2013).

#### CRH and its relationship to immune therapies

Drugs have been proposed to either block CRHR-1 and CRHR-2 receptors as CRH inhibitors with therapeutic properties through modifying the immune response as tool for both CNS and inflammatory disorders associated with central and peripheral CRH. CRH-R1 antagonist could be considered for the treatment of allergic conditions, such as urticaria, asthma, eczema, or disorders that increase blood–brain barrier permeability (Theoharides et al., 1998; Esposito et al., 2002), chronic inflammatory bowel syndromes, irritable bowel disease, and ulcerative colitis and other pathologies (Kawahito et al., 1995; Gravanis and Margioris, 2005).

**Figure 2:**
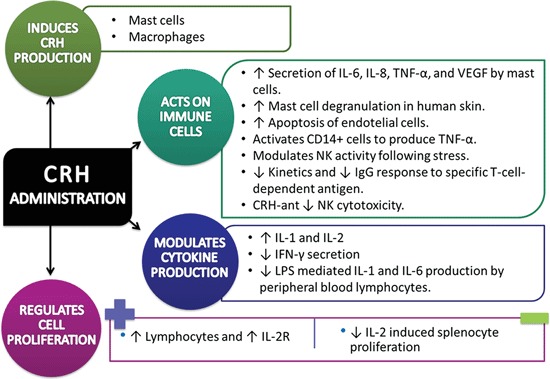
**Experimental data of Corticotropin-Releasing Hormone/Corticotropin-Releasing Hormone agonist impact on immune system cells and processes (↑= increase, ↓= decrease).**

The effects of CRH administration on immune system are summarized in Figure 2.

#### Future directions

There are two major problems associated with the HPA axis. The inflammatory process and the reduction in the immune response. Chronic exposure to antiinflammatory steroids have effects such as immunosuppression which under certain conditions can cause undesired effect. Identifying specific receptor subtypes to CRH, or applying interleukin receptor blockers may provide a viable alternative to maintaining of the homeostatic mechanisms.

Emotional stress causes somatic alterations including the immune response and is perhaps, one of the most pressing problems in global public health that needs to be addressed.

## Gonadotropin-Releasing Hormone (GnRH)

GnRH, also known as luteinizing hormone releasing hormone (LHRH), is a small peptide hormone (pGlu-His-Trp-Ser-Tyr-Gly-Leu-Arg-Pro-Gly-NH_2_) of 1.2 kDa, with an established role as central regulator of the neuroendocrine reproductive axis in both males and females (Cheung and Wong, 2008).

This neuropeptide is mainly synthesized in hypothalamic neurons of the medial preoptic area. It is well established that adenohypophysial gonadotropic cells stimulate the synthesis and secretion of gonadotropic hormones such as follicle stimulating hormone (FSH) and luteinizing hormone (LH) (Cheng and Leung, 2005). LH and FSH are released into the systemic circulation in a pulsatile pattern, which is dependent upon the GnRH pulse frequency, and act to affect gonadal steroidogenesis and gametogenesis (Sasaki and Norwitz, 2011).

### GnRH isoforms

At the present, it is known that, in addition to the hypothalamic GnRH, several other isoforms of the decapeptide exist in vertebrates. All these isoforms are characterized by the conservation of the length of the peptide as well as by the amino acid sequences of the N-terminal (Glp-His-Trp-Ser) and C-terminal (Pro-Gly-NH_2_) domains, indicating that these molecular features are critical for the binding to, and the activation of, the receptor (Limonta and Manea, 2013). The classical hypophysiotropic GnRH located in the anterior hypothalamus/preoptic area is also designed as GnRH-I (Stevenson et al., 2012).

A second isoform of GnRH, originally isolated from the chicken hypothalamus and referred to as chicken GnRH or cGnRH, has been isolated in most vertebrate species—including humans—is now referred to as GnRH-II and is 70% homologous to GnRH-I (Sasaki and Norwitz, 2011). GnRH-II is expressed at a higher level outside of the brain (Tan and Bukulmez, 2011).

A third GnRH isoform, GnRH-III, was isolated from sea lamprey and was also detected in the brain of mammals (Tan and Bukulmez, 2011). This isoform has 60% homology with GnRH-I. In the lamprey, GnRH-III is localized in brain areas involved in the control of reproductive functions and it has been reported to stimulate steroidogenesis and gametogenesis, but it has 500–1000 times less potency in LH and FSH secretion both *in vitro* and *in vivo* (Limonta and Manea, 2013).

GnRH is small and has good thermal and chemical stability and no internal Cys bridges (minimal fixed structure). Its size and stability has led to the availability of thousands of analogs (Conn and Janovick, 2009).

### Synthesis and secretion

GnRH is synthesized as a prohormone, human preproGnRH that contains 92 amino acids (Harrison et al., 2004). The gene encoding preproGnRH1 gene has been cloned in number of species and is approximately 4300-bp with four relatively short exons separated by three large introns (Clarke and Pompolo, 2005).

Neurons that synthesize and release GnRH (GnRHergic neurons) have their origin in embryonic olfactory placodes. These embryonic cells, migrate and colonize the basal forebrain, preoptic area around the basal and medium hypothalamus. The median eminence in mammals contains a large amount of GnRH, so is the area in which the peptide is stored in neural terminals before release into the hypothalamus-pituitary portal system (Clarke and Pompolo, 2005).

GnRH is released as synchronized pulses from the nerve endings of about 1000 neurons into the hypophyseal portal system every 30–120 mins to stimulate the biosynthesis and secretion of LH and FSH from gonadotrophs (Millar, 2005). In adult rodents, GnRH is released from the median eminence at a frequency of about one pulse every 30 mins. A slightly slower frequency of release (approximately 50–60 mins intervals) is observed in primates (Yin and Gore, 2006). On the other hand, continuous (non-pulsatile) GnRH administration or long-lasting GnRH-a desensitize GnRHR, thereby decreasing/inhibiting the release of LH and FSH by the pituitary (Wilson et al., 2007).

### Half-life

GnRH half-life normally is about 2–4 mins (Walters et al., 2008). Its short half-life is because of the rapid cleavage of the bonds between amino acids at positions 5–6, 6–7, and 9–10. Substitution at position 6 in GnRH agonists (GnRH-a) yields them resistant to degradation and increases its half-life and the time of receptor occupancy (Magon, 2011).

### GnRH receptor (GnRHR)

The human pituitary GnRHR belongs to the family of GPCRs and is one of the smallest GPCRs (328 amino acid protein in the human); it may contain only the essentials required for ligand binding and signal transduction (Conn and Janovick, 2009).

Distinct forms of the GnRHR can be broadly divided into two groups:

1. The type I GnRH-Rs have greater affinity for GnRH-I than for GnRH-II and lacks a typical intracellular COOH terminus; this group contains all known mammalian GnRH-Rs except for the recently described primate type II GnRH-R (McArdle et al., 2002; Ciechanowska et al., 2010).
2. Type II GnRH-Rs have greater affinity for the GnRH-II and possess C-terminal tails of varying length; this group contains all the known receptors from non-mammalian vertebrates (catfish, goldfish, chicken, goldfish, *Xenopus*, etc.) in addition to the type II primate GnRH-Rs (McArdle et al., 2002).

### Extrahypothalamic and extrapituitary location of GnRH and GnRHR respectively

#### GnRH outside the pituitary zone

GnRH has been located in several extrahypothalamic cells and tissues: GnRH-like substances have been reported to be secreted from tissues outside the CNS of rats and humans. Particularly, a (GnRH)-like protein has been detected in human ovaries (Aten et al., 1987; Dong et al., 1993; Peng et al., 1994). Also, Oikawa demonstrated by PCR amplification of cDNA from rat hypothalamus, granulosa cells, and whole ovary, a 241-bp product identical to the authentic GnRH sequence based on analysis on both strands (Oikawa et al., 1990).

RT-PCR analysis together with Southern blot analysis demonstrated the presence of GnRH mRNA in human testes and mammary gland (Dong et al., 1993).

Furthermore, the gene coding for GnRH is expressed in immune tissue and immunoreactive GnRH is measurable in immune cells. Analysis of mononuclear cells by RT-PCR coupled to Southern hybridization of total RNA confirmed that GnRH genes are expressed in human peripheral blood lymphocytes. The GnRH transcripts in mononuclear cells were identical to the hypothalamic GnRH and PRL release inhibiting factor cDNA, at least within this 380-bp fragment (Chen et al., 1999).

Rat splenocytes contain an immunoactive and bioactive GnRH (Emanuele et al., 1990; Azad et al., 1991) that has also been found in lymphocytes from rat thymus (Marchetti et al., 1990; Maier et al., 1992). Moreover, it has been reported that normal and leukemic human T cells produce GnRH-II and GnRH-I and that the incubation of those cells with these neuropeptides triggered *de novo* gene transcription and cell-surface expression of a 67-kDa non-integrin laminin receptor that is involved in cellular adhesion and migration and in tumor invasion and metastasis (Chen et al., 2002).

The fact that recent evidence shows the existence of diverse isoforms of GnRH, with high sequence homology and a core variable region raises the issue that previous GnRH distribution studies may have identified a variety of isoforms. To confirm the distribution of GnRH-I only, Khan et al. (2003), reported that intense GnRH-I immunoreactivity was found in Kupffer cells, and less intense in spleen lymphocytes and follicular dendritic cells in rat.

Other cells that have been reported to express GnRH and its mRNA are mast cells from the nervous system and the peritoneal cavity, as detected by immunohistochemistry and RT-PCR, respectively (Silverman et al., 1994; Gill and Rissman, 1998; Khalil et al., 2003).

#### Extrapituitary GnRHR

GnRH binding sites have been described throughout the CNS and PNS as in several non-CNS cells and tissues:

Human ovarian granulosa-luteal cells also express the GnRHR mRNA (Chen et al., 1999).

GnRHR expression in organs and cells of the immune system has been confirmed: GnRH binding sites have been reported in rat spleen (Batticane et al., 1991) and thymus, thus potentially enabling GnRH-a to have direct stimulatory effects that might contribute to improved T-cell function (Marchetti et al., 1989; Batticane et al., 1991; Morale et al., 1991).

RT-PCR coupled to Southern hybridization of total RNA obtained from mononuclear cells confirmed that the GnRHR is expressed in human peripheral blood lymphocytes (Chen et al., 1999) as in porcine lymphocytes (Standaert et al., 1992).

Chen and colleagues reported that the GnRHR transcript expressed in mononuclear cells has an identical nucleotide sequence as the pituitary GnRHR cDNA. In this work, a time-course study of the effect of GnRH on mononuclear cells was performed and it was found that GnRHR mRNA increased gradually after *in vitro* culture, and this increase was further augmented by treatment with GnRH for up to 24h. In contrast, the expression of GnRH mRNA was decreased by GnRH treatment in a dose-dependent manner and the IL-2R**γ** subunit gene was highly expressed in mononuclear cells. At this point we can speculate that these GnRH-binding sites in lymphocytes are probably functional, as many studies have demonstrated that GnRH-a caused a dose-dependent increase in LH production, whereas GnRHR antibodies blocked this action (Chen et al., 1999).

It has also been shown that blockade of central and peripheral GnRHR with a potent GnRH antagonist (GnRH-ant) during a critical period of maturation can impair the morphological development of thymus and the cellular and humoral immune responses in rats (Morale et al., 1991) and monkeys (Mann et al., 1994).

Particularly, neonatal exposure of male primates to a GnRH-ant alters early postnatal programming of immune function by reducing the number of B cells and T cells in the thymic medulla and T cells in the periarterial lymphatic sheaths of the spleen. GnRH-ant treatment increased the frequency of clinical problems, lowered circulating levels of lymphocytes, total T cells, CD8+ T cells and B cells, and altered the proliferative index of lymphocytes to mitogens. As adults, the cell- and humorally-mediated immune responses remained impaired (Mann et al., 2000).

To evaluate regions of GnRH-I binding activity, a biotinylated GnRH-I sequence was used with avidin-labelled HRP, and results showed GnRH-I binding activity present in Kupffer cells, spleen lymphocytes and follicular dendritic cells, as well as in thymocytes and peripheral blood lymphocytes and neutrophils (Khan et al., 2003).

### GnRH and immune system

GnRH and sex steroids seem to be important components of immune system modulation and may play a role in the regulation of immune mediated diseases. A comprehensive description of such interactions has been reviewed by Tanriverdi et al. (2003) and Quintanar (2011).

#### Immune response to GnRH administration

##### Proliferation

###### Induction

When splenocytes and thymocytes are preincubated with GnRH-a or GnRH peptide, an augmentation of the proliferative response is obtained. Marchetti et al. (1989) showed a significant dose-dependent increase on proliferative response to the mitogen ConA in rat thymocytes. This response was abolished with simultaneous addition of a GnRH-ant. Also, splenocytes and thymocytes from proestrus female rats incubated with GnRH or its analogs, significantly increased the basal proliferative activity which was accompanied by a specific increase in IL-2 receptor-positive cells (Batticane et al., 1991).

Furthermore, *in vivo* GnRH-a treatment prevents thymus atrophy and markedly stimulates thymocyte blastogenic activity in hypophysectomized rats, thus suggesting that GnRH and its agonistic analogs are may capable to exert a direct modulation of immune system function (Marchetti et al., 1989).

Injection of GnRH-a increases CD8+ T-cell levels and proliferation to a variety of mitogens (Mann et al., 1994). In another study, it has been reported that GnRH immunization in rats induced the presence of eosinophils in intertubular tissue of testes whereas in unimmunized controls these cells were not present (Duckett et al., 1997).

Moreover, in a rat model of pregnancy-induced thymic involution, GnRH-a infusions markedly attenuated pregnancy-induced thymic involution resulting in significant increases in thymic weight and thymocyte numbers (Dixit et al., 2003a).

In the same way, Sutherland et al. (2008) made a pilot study in patients, where a GnRH-a (goserelin) was given prior to allogeneic or autologous hematopoietic stem cell transplantation. GnRH-a administration significantly increased neutrophil and lymphocyte numbers within the first month of posttransplantation indicating that either directly or indirectly, GnRH induced an increase in circulating neutrophils.

It has been reported that treatment with the GnRH-a, leuprolide acetate (50 μg/mouse, s.c.) prior to restraint stress, significantly prevented its effect on cell-mediated immunity, antibody titer levels, total leukocyte count and relative thymus weight, showing a significant increase of these immunological parameters (Umathe et al., 2008).

###### Supression

To test whether a GnRH-a could alter *in vivo* human immune cells, Ho et al. (1995) treated a group of infertile patients under a protocol of buserelin acetate administration and peripheral B cells, NK cells, CD4+ and CD8+ T cells, and the expression of CD69, CD25, human leucocyte antigen-DR (HLA-DR), and CD71 antigens on the T cells were serially examined by dual-color flow cytometry. They found that the GnRH-a had a transiently immunosuppressive effect on CD4+ and CD25+ T cells, but CD69+, CD25+, and HLA-DR+ T cells were activated during and after successful implantation. Regarding to the B cells, NK cells, CD8+ T cells, and CD71+ T lymphocyte subpopulations there were no changes throughout the whole course of treatment.

On the other hand, several groups have provided evidence that the *in vitro* proliferation of a variety of human ovarian, endometrial, and breast cancer cell lines can be inhibited by agonistic and/or antagonistic analogs of GnRH in a dose- and time-dependent manner (Eidne et al., 1987; Thompson et al., 1991; Emons et al., 1993a,b; Emons and Schally, 1994).

In ovarian cancer cells, GnRH seems to have two opposite activities:

1. The antimitotic activity through inhibition of signal transduction of mitogens such as epidermal growth factor, and
2. The inhibition of Doxorubicin-induced apoptosis via activation of transcription nuclear factor kappa B (NFkB) as shown by Gründker et al. (2000). In this report is clearly suggested that GnRH-a Triptorelin inhibits Doxorubicin-induced apoptosis via activation of NFkB.

Furthermore, biopsy specimens collected from lesions, myometria and corresponding endometria of women with ovarian endometrioma, adenomyosis and uterine myoma treated with the GnRH-a, leuprolide acetate, for a variable period of 3–6 months before surgery, showed a significantly decrease in the infiltration of CD68-positive Mφ, micro-vessel density and tissue levels of monocyte chemotactic protein I (MCP-I). Furthermore, when compared with the non-treated group, a significant increase in apoptotic index and quantitative-histogram scores of activated caspase-3 after GnRH-a therapy were observed in the eutopic endometria, lesions and myometria of these diseases (Khan et al., 2010).

Tanriverdi et al. (2005) demonstrated that GnRH-I and GnRH-II have differential modulatory effects on human PBMC proliferation and IL-2R*γ*-chain mRNA expression in healthy males. Treatment of *in vitro* PMBCs with GnRH-I and with IL-2 resulted in a significant increase in cell proliferation as well as in an increased expression of IL-2R*γ* mRNA in a dose dependent manner compared with the untreated control. In contrast, GnRH-II administration did not affect the proliferation of PMBCs alone, and IL-2R*γ* expression was significantly decreased in all concentrations. This study clearly indicates the potential stimulatory effects of GnRH-I in PBMCs and reduction of these effects by GnRH-II.

##### Induces GnRH production by immune cells.

Numerous interesting and solid studies show that T-cells (and other immune cells) can in fact produce and secrete various endogenous neurotransmitters, either spontaneously or after induction by external stimuli. Among neurotransmitters produced by T-cells are GnRH-I and GnRH-II (Levite, 2008).

Immunoactive and bioactive GnRH peptide is present and the GnRH message is expressed in human peripheral blood T-cells. Both CD4^+^ and CD8^+^ subsets of T-cells produce GnRH and it is significantly increased when cells are activated by either phytohemagglutinin or anti-CD3 antibody. This GnRH production in activated T-cells is an early activation event that is independent of IL-2 system activation and DNA synthesis (Azad et al., 1993).

##### Induces immune mediators production

To investigate the role of GnRH in the modulation of T helper cytokines in pregnant rats undergoing termination of pregnancy, Dixit et al. (2003b) administrated a GnRH-a to evaluate effects on Th-1 and Th-2 cytokines. A marked increase in IFN-*γ*, inhibition of IL-4 production and an IL-2 and IL-10 absent response was observed. It is possible that IL-2 levels are unchanged because it might has been consumed by activated T cells (Quintanar, 2011).

Recently, Goldberg et al. (2009) demonstrated in a clinically relevant model of allogeneic bone marrow transplantation, that after leuprolide acetate treatment, the enhanced peripheral T cell reconstitution is due to both increases in lymphoid-committed precursors as well as enhanced thymic regeneration.

GnRH has also been reported to regulate four angiogenic chemokines expression (CXCL2, CXCL3, CXCL6, and CXCL8) in human placenta, where trophoblasts were subsequently shown to recruit lymphocytes. Additionally, in Jurkat T cells and primary peripheral blood T and uterine NK cells; chemokine release was detected in chemotaxis assays and this was shown to be GnRH dependent (Cavanagh et al., 2009).

There are two works evaluating the efficacy of the GnRH-a, leuprolide acetate, on NK cells activity in endometriosis. Results are contradictory since the first one suggests an increased NK cell activity in peripheral blood samples determined by 51Cr release assay (Umesaki et al., 1999); and the second one reported that NK cell cytotoxicity from control and patients was significantly decreased with leuprolide acetate (Wong and Simon, 2004). These findings suggest a direct immunomodulatory role of GnRH on NK cell activity.

In an attempt to know the possible immunomodulatory effects of GnRH on LPS activated human peripheral blood monocytes cultured *in vitro*, Komorowski and Stepień (1995) found that GnRH did not affect the secretion of the cytokines IL-1β and IL-6.

On the other hand, there is a report where the immunomodulation exerted by GnRH on freshly isolated primary peritoneal macrophages is clearly observed. In this work the authors found that the production of nitric oxide, co-stimulated with LPS and IFN*γ*, and the activity of NF-kB was suppressed by GnRH exposure. Taken together, these results demonstrate that GnRH participates in macrophage function and indicate that the NF-kB signaling pathway may be responsible for GnRH-mediated immune modulation (Min et al., 2009).

#### GnRH expression response against immune system challenges

There is a work that reported that when lymphocyte lysates were applied to rat anterior pituitary cells in monolayer culture, significant stimulation of GnRH secretion was seen. This response evoked by lymphocyte lysates was found to be dose dependent and could be significantly inhibited by a GnRH-ant (Emanuele et al., 1990).

Otherwise, TNF has a number of regulatory actions on gonadotrophic functions: TNF and other cytokines, notably IL-1β, have powerful LH suppressing abilities on pituitary activity (Kalra et al., 1998).

#### GnRH and autoimmunity

Given that GnRH possesses direct immunostimulatory actions, it has been hypothesized that GnRH might play a role in the development of autoimmune disease.

Jacobson et al. (1994) demonstrated that the administration of GnRH antagonists in a mouse model of systemic lupus erythematosus (SLE), led to a reduction in autoantibody levels, total immunoglobulin levels, renal disease, and significantly improved survival, independently of gonadal steroids.

Based on the above mentioned findings and considering that (1) in the nonobese mouse model of autoimmune diabetes (NOD mouse), castration of male NOD mice leads to increased incidence of diabetes, and that (2) castration of male mice leads to an increase in GnRH action, Ansari et al. (2004) determined the effect of GnRH agonists and antagonists on expression of autoimmune diabetes in this mouse model. Their results showed that Antide administration prevented the increased incidence of diabetes, reduced total serum IgG levels, IL-6 cytokine expression in cultured splenocytes, and the lymphocytic infiltration of islets. GnRH administration exerted reciprocal effects, leading to earlier timing of onset of diabetes and increased serum total IgG levels.

In addition, due to that has been reported that motoneurons of spinal cord of rat from embryonic until adult stage, express GnRHRs which respond to GnRH (Quintanar et al., 2009) a possible clinical use in spinal cord injury (Calderón-Vallejo and Quintanar, 2012) and CNS autoimmune diseases has been proposed. Specifically, treatment with GnRH (Quintanar, 2011) or a synthetic analog (Guzmán-Soto et al., 2012) induces an improvement of clinical signs in the EAE model, where they remained significantly lower in EAE rats with GnRH administration compared to animals without treatment. In these experiments, also a significantly increased expression of neurofilaments (NFs) and myelin basic protein as well as more axons of larger areas in the spinal cord of EAE animals were found.

A great advantage of GnRH and its analog leuprolide acetate, is that they are capable to cross the blood brain barrier (Kastin et al., 1980; Barrera et al., 1991) and reach their targets into the CNS. Taking into account this advantage of leuprolide acetate and its effect on recovery in locomotion and increased expression of biological markers, the use of this agonist as a potential therapeutic approach for traumatic or neurodegenerative diseases such as multiple sclerosis should be considered.

**Figure 3:**
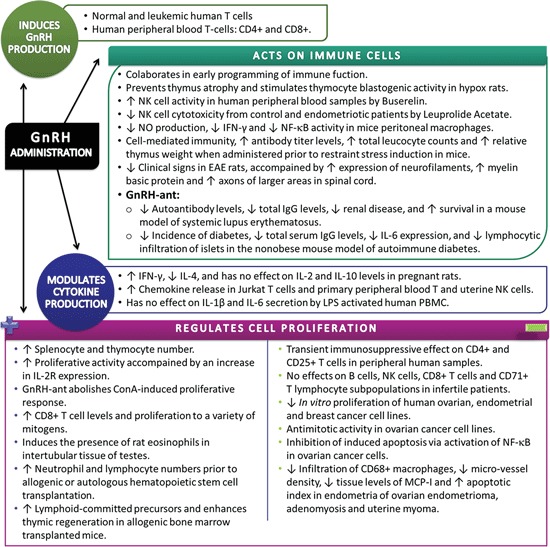
**Summary of experimental data showing the effects of Gonadotropin-Releasing Hormone/Gonadotropin-Releasing Hormone agonist on the immune system**. Extrapituitary GnRH is implicated in a wide range of immunological processes, including direct cell immunomodulation and proliferative responses (↑= increase, ↓= decrease).

The effects of GnRH on immune system are summarized in Figure 3.

#### Clinical relevance and future directions

GnRH induces the secretion of FSH-LH and consequently of sex hormones, which are involved in the immune response. Controversial is the idea that sex hormones are immunoprotective because depending on the type of hormone and experimental conditions can be obtained opposite effects.

The presence of GnRH receptors and GnRH secretion by cells of the immune system, opening the spectrum of possibilities for interaction of autocrine or paracrine regulation. However, the pharmacological use of GnRH or its analogues, independently of the direct effect of sex hormones, raise the possibility that can be used as a inflammatory regulator in autoimmune diseases. Retrospective studies can be analyzed to establish whether treatment with GnRH analogues have improved immunological conditions of patients with autoimmune pathology. This option can be explored in future works.

## Conclusion

Hypothalamic hormones are closely related to the immune system. Their direct and indirect effects establish a bidirectional network to establish the required mechanisms to maintain homeostasis.

TRH, CRH and GnRH exhibit common patterns of specific receptors in immune cells and tissues. It is hard to think in the action of a single independent factor within the neuroimmunoendocrine network. When any of these elements destabilize, the system is altered and is difficult to define which factor of all may have been responsible for the alteration. Perhaps because of the complexity of the network, experiments to demonstrate such events become complicated to study. Using *in vitro* models allow clarify in a specific manner some of the elements involved in the homeostasis, however, extrapolate it to *in vivo* models could lead to unreliable interpretations.

In the evolution of the knowledge of the involvement of neurohormones on the immune system, we can find that many of the investigations are related to their pharmacological application, however, few of them concern to the physiological mechanism of its immunomodulation either autocrine or paracrine. If more is known about the physiology of these neurohormones, expectations about their applications at therapeutic level or to the understanding of our complex responses to internal or external challenges to maintain the survival of the individual could be proposed.

## Conflict of interest statement

The authors declare that the research was conducted in the absence of any commercial or financial relationships that could be construed as a potential conflict of interest.
